# Supplementary material for: Bending forces and nucleotide state jointly regulate F-actin structure
Source: Nature. 2022 Oct 26;611(7935):380–6. doi: 10.1038/s41586-022-05366-w (PMC9646526; doi:10.1038/s41586-022-05366-w)
Supplement: Supplementary file 1 — Supplementary Tables 1–3: cryo-EM data collection, refinement and validation statistics, and twist travelling wave equation parameters. Supplementary Discussion: detailed structural analysis and thermal fluctuation modelling of bending deformations. [file 41586_2022_5366_MOESM1_ESM.pdf]

nature portfolio

https://doi.org/10.1038/s41586-022-05366-w

Supplementary information

Bending forces and nucleotide state jointly regulate F-actin structure

In the format provided by the authors and unedited

Supplementary Table 1. High-resolution cryo-EM data collection, refinement, and validation. statistics. Cryo-EM data collection, refinement, and validation statistics for helical ADP-F-actin helical and ADP-P_i-F-actin.

Supplementary Table 2. Instantaneous twist traveling wave equation parameters.
The modelled parameters for the traveling wave equation. Uncertainties represent one standard deviation error estimates of parameter fits.

Supplementary Table 3. Asymmetric cryo-EM data collection, refinement, and validation statistics.
Cryo-EM data collection, refinement, and validation statistics for two independently-processed, straight, asymmetric ADP-F-actin reconstructions, the asymmetric bent ADP-F-actin reconstruction, and the asymmetric ADP-P_i-F-actin reconstruction.

Supplementary Discussion

Supplementary Table 1. High-resolution cryo-EM data collection, refinement, and validation.

	ADP-F-actin (EMD-27114, PDB 8D13)	ADP-P_i-F-actin (EMD-27115, PDB 8D14)
**Data collection and processing**		
Microscope	Titan Krios	Titan Krios
Voltage (kV)	300	300
Detector	K2 Summit	K2 Summit
Magnification	29,000	29,000
Electron exposure ($e^{-}Å^{-2}$)	60	60
Exposure rate (e^{-}/pixel/s)	6	6
Calibrated pixel size (Å)	1.03	1.03
Defocus range (µm)	–1.5 to –3.5	–1.5 to –3.5
Symmetry imposed	C1<br>28.07 Å rise<br>–166.69° twist	C1<br>27.81 Å rise<br>–166.64° twist
Initial particle images (no.)	220,462	611,403
Final particle images (no.)	219,569	374,778
Map resolution (Å)	2.43	2.51
FSC threshold	0.143	0.143
Map sharpening B factor ($Å^2$)	–32.1	–41.2
Map resolution range (Å)	2.40-3.42	2.50-3.16
**Refinement**		
Initial models (PDB ID)	7R8V	7R8V
Model resolution (Å)	2.46	2.56
FSC threshold	0.5	0.5
Model resolution range (Å)	2.41-3.07	2.50-3.16
Model composition	3 actin protomers	3 actin protomers
Non-hydrogen atoms	9216	9255
Protein residues	1113	1113
Ligands	3 Mg^{2+}, 3 ADP, 432 water	3 Mg^{2+}, 3 ADP, 3 PO_4^{3-}, 426 water
B factors ($Å^2$)		
Protein	61.15	66.09
Ligand	22.18	21.73
Water	35.34	39.46
R.M.S. deviations		
Bond lengths (Å)	0.004	0.004
Bond angles (°)	1.008	0.986
Validation		
MolProbity score	1.20	1.31
Clashscore	4.19	5.73
Poor rotamers (%)	0.64	0.32
Ramachandran plot		
Favored (%)	99.18	98.91
Allowed (%)	0.82	1.09
Disallowed (%)	0.00	0.00
EMRinger Score	5.17	5.68

Supplementary Table 2. Instantaneous twist traveling wave equation parameters.

Parameter symbol	Units	Parameter physical description	ADP-F-actin Strand 1	ADP-F-actin Strand 2	ADP-P_i-F-actin Strand 1	ADP-P_i-F-actin Strand 2
A	° µm	Twist-curvature coupling factor	26.83 ± 0.46	-26.59 ± 0.50	19.53 ± 0.32	-19.82 ± 0.40
k	Radians µm	Propagation factor	3.45 ± 0.16	3.55 ± 0.17	3.46 ± 0.43	3.46 ± 0.42
ω	Radians / position	Period of twist	0.246 ± 0.003	0.246 ± 0.004	0.245 ± 0.004	0.238 ± 0.005
φ	Radians	Phase shift of twist	3.29 ± 0.09	3.24 ± 0.10	3.21 ± 0.18	3.30 ± 0.19
B	°	Average twist	-166.65 ± 0.17	-166.81 ± 0.19	-166.57 ± 0.13	-166.46 ± 0.19

Supplementary Table 3. Asymmetric cryo-EM data collection, refinement, and validation statistics.

	Straight ADP-F-actin 1 (EMD-27118, PDB 8D17)	Straight ADP-F-actin 2 (EMD-27119, PDB 8D18)	Bent ADP-F-actin (EMD-27116, PDB 8D15)	Bent ADP-P_i-F-actin (EMD-27117, PDB 8D16)
**Data collection and processing**				
Microscope	Titan Krios	Titan Krios	Titan Krios	Titan Krios
Voltage (kV)	300	300	300	300
Detector	K2 Summit	K2 Summit	K2 Summit	K2 Summit
Magnification	29,000	29,000	29,000	29,000
Electron exposure ($e^{-}Å^{-2}$)	60	60	60	60
Exposure rate (e^{-}/pixel/s)	6	6	6	6
Calibrated pixel size (Å)	1.03	1.03	1.03	1.03
Defocus range (μm)	–1.5 to –3.5	–1.5 to –3.5	–1.5 to –3.5	–1.5 to –3.5
Symmetry imposed	C1	C1	C1	C1
Initial particle images, with segment overlap (no.)	138,415	138,415	77,559	124,176
Final particle images, no overlap within mask (no.)	7,833	7,833	10,753	14,991
Map resolution (Å)	3.69	3.66	3.61	3.71
FSC threshold	0.143	0.143	0.143	0.143
Map sharpening B factor ($Å^2$)	-47.8	-56.0	–64.6	-50.0
Map resolution range (Å)	3.44-10.65	3.50-10.68	3.51-10.05	3.52-8.98
**Refinement**				
Initial models (PDB ID)	8D13	8D13	8D13	8D14
Model resolution (Å)	3.63	3.65	3.65	3.70
FSC threshold	0.5	0.5	0.5	0.5
Model resolution range (Å)	3.44-5.87	3.50-5.89	3.51-5.53	3.52-5.89
Model composition	7 actin protomers	7 actin protomers	7 actin protomers	7 actin protomers
Non-hydrogen atoms	20,496	20,496	20,496	20,531
Protein residues	2597	2597	2597	2597
Ligands	7 Mg^{2+}, 7 ADP	7 Mg^{2+}, 7 ADP	7 Mg^{2+}, 7 ADP	7 Mg^{2+}, 7 ADP, 7 PO_4^{3-}
B factors ($Å^2$)				
Protein	76.50	81.02	61.54	87.28
Ligand	43.55	40.50	28.33	42.44
R.M.S. deviations				
Bond lengths (Å)	0.005	0.005	0.005	0.011
Bond angles (°)	0.664	0.680	0.678	0.708
Validation				
MolProbity score	1.70	1.76	1.78	1.88
Clashscore	6.12	6.66	6.07	7.54
Poor rotamers (%)	0.05	0.00	0.05	0.00
Ramachandran plot				
Favored (%)	94.70	94.15	93.10	92.62
Allowed (%)	5.30	5.85	6.90	7.38
Disallowed (%)	0.00	0.00	0.00	0.00
EMRinger Score	3.30	2.99	3.15	2.71

Supplementary Discussion

Comparison with previous molecular dynamics simulations

The role of solvent in F-actin has previously been explored through molecular dynamics simulations[42]. This work, which focused on ATP-F-actin, found that the presence of explicit solvent stabilized the F-actin subunit conformation. We observe similar distances in our ADP-P_i-F-actin structure (~4 Å) between the key active residues D11, D154, and K18 and the bound magnesium ion, consistent with this report. The positioning of active site water molecules nevertheless appears to be distinct in our experimental structure of ADP-P_i-F-actin, suggesting that our structures (as well as those of Raunser and colleagues[39]) will likely be valuable for guiding future molecular dynamics studies of F-actin's ATPase mechanism.

Detailed analysis of water-mediated inter-subunit contacts

Lateral interactions occur between the D-loop of subunit i, H-plug of subunit i+1, and a surface on subdomain 3 of subunit i+2 which we refer to as "site 1". We observe only a single direct side-chain mediated interaction across strands through H-plug residue E270; however, 6 bridging waters, many of which are coordinated by backbone carbonyls, span the interface (Fig. 2b, top). While there is minor repositioning of waters between the ADP and ADP-P_i states, their number and coordination geometry is preserved.

Longitudinal interactions occur across an extensive interface between subunit i and subunit i+2, which has previously been predicted to contain two divalent cation binding sites: a buried "polymerization" site and a more exposed "stiffness" site within subdomain 2[43]. A bi-partite major interface is formed between subunit i's subdomain 2 and a surface of subunit i+2's subdomain 3 which we refer to as "site 2". The D-loop forms several contacts, none of which we found to be solvent-mediated. This interface overlaps with the "stiffness" cation binding site, which we found to be unoccupied. However, this may be a limitation of local resolution, as the density maps in this region did not feature sufficient detail to confidently model solvent molecules.

We also observe an extensively hydrated interface on the surface of subdomain 2's core, containing four bridging waters in the ADP-P_i state and one additional water in the ADP state, that features only a single direct side-chain mediated salt bridge. This interface overlaps with the "polymerization" cation binding site, and two of the density peaks we have modelled as waters could feasibly be cations (which we cannot discriminate in our density maps). In particular, the water bridging subunit i residue T202 and subunit i+2 residue D286 (ADP-F-actin water C 438 / ADP-P_i-F-actin water C 482 in the deposited models) is exactly positioned in the predicted site, while another water (ADP-F-actin B 443 / ADP-P_i-F-actin B 411) is closely adjacent.

A minor interface is also formed between subunit i's subdomain 4 "flap" and subunit i+2's subdomain 3 site 1 (the same site which mediates lateral interactions), once again featuring a single side-chain mediated salt bridge, as well as two bridging waters which are equivalently positioned in the ADP and ADP-P_i states. In summary, we find that both lateral and longitudinal interfaces are extensively solvated, which we hypothesize could lubricate mechanical rearrangements within the filament.

Thermal fluctuation modelling of curvature distributions

The curvature distribution of free, unloaded actin filaments would be expected to follow a Boltzmann distribution defined by F-actin's persistence length and the filament segment length. Specifically, the energy to bend an actin filament is given by:

$$E = \frac{1}{2} k_B T \, L_p \, L \, \kappa^2$$

Where k_B is the Boltzmann constant, T is absolute temperature, L_p is persistence length in microns, L is segment length in microns, and κ is segment curvature in inverse-microns[37]. In an F-actin system at thermal equilibrium, the probability of a filament segment having a given curvature ($P(\kappa)$) is given by the following equation:

$$P(\kappa) = \frac{1}{Z} e^{\frac{-L_p \, L \, \kappa^2}{2}}$$

Where the partition function Z is the sum of all of the Boltzmann factors for each curvature.

$$Z = \sum_{k=0}^{\infty} e^{\frac{-L_p \, L \, \kappa^2}{2}}$$

Using the measured persistence lengths[35] of 9 μm for ADP-F-actin and 11 μm for ADP-P_i-F-actin, and setting a constant segment length of 500 Å (roughly the end-to-end length of a 16-protomer filament, as was reconstructed for cryoDRGN analysis), the expected Boltzmann distributions for ADP- and ADP-P_i-F-actin were modelled (Extended Data Fig. 4a). These Boltzmann models roughly fit the distributions, and they demonstrated that increasing persistence length corresponds to a narrowing of the curvature distribution, with fewer segments having high curvature, consistent with our observation that the ADP-F-actin dataset had a higher proportion of segments featuring high curvature (Extended Data Fig. 4b, Fig. 3b).

However, analysis of the residuals between the model and measured curvature distributions revealed non-random deviations from zero, suggesting a systematic difference between the model and data. To assess whether deviations could be a result of persistence length measurement inaccuracies, the probability function was adjusted to include a multiplicative factor in the energy term, α:

$$P_{\text{adjusted}}(\kappa) = \frac{1}{Z_{\text{adjusted}}} e^{\frac{-\alpha L_p L \kappa^2}{2}}$$

$$Z_{\text{adjusted}} = \sum_{k=0}^{\infty} e^{\frac{-\alpha L_p L \kappa^2}{2}}$$

Fitting that parameter to the distributions (Extended Data Fig. 4a) reduced the magnitude of the residuals overall, but it did not eliminate the systematic deviations. This suggests that the data are not perfectly modelled by the simple Boltzmann distribution and the deviations cannot be explained entirely by inaccuracies in the persistence length estimates. One likely cause could be that external force imposed on the filaments due to fluid flow would increase the population of high-curvature filament segments. It is also possible that a more sophisticated model independent of constant filament segment length assumptions and that computes bending energy by integration along entire filaments may be able to account for these deviations.

Furthermore, this modelling reveals that the reconstructed cryoDRGN volumes had bending energies in the range of ~1-7 $k_B T$, consistent with bending deformations of this scale being associated with active bending[9,10]. However, these estimates are limited by the accuracy of persistence length measurements and the accuracy of the elastic rod bending energy approximations at the discrete molecular scale.

Coarse-grained description of F-actin bending transitions

To characterize bending-dependent lattice deformations in detail, we examined the relative repositioning of subdomains of neighboring protomers along the lattice (Extended Data Fig. 6). The most notable systematic deviations were the subdomain 1-subdomain 1, subdomain 4-subdomain 4 (Extended Data Fig. 5c, top row), and subdomain 2-subdomain 1 distances, as well as their opposing angles (Extended Data Fig. 5c, middle row). These changes are consistent with the prediction of architectural remodelling in bent F-actin derived from early negative stain electron microscopy studies[47]. This suggests longitudinal contacts are deformed by filament bending. Inter-strand angles also separated by the reference strand used for making each comparison, suggesting the filament core water channel and lateral contacts are additionally deformed. These observations are consistent with a lubricating role for bridging waters at inter-subunit contacts. Bent ADP-P_i-F-actin furthermore exhibited shorter average inter-subdomain distances and smaller angles, suggesting the slightly smaller average ADP-P_i-F-actin rise could prime the filament to differentially sample F-actin's bending conformational space. Notably, bent F-actin in both nucleotide states exhibited no clear systematic differences in the intra-subunit subdomain 2-subdomain 1-subdomain 3-subdomain 4 dihedral angle, the major parameter describing changes in

subunit flattening, suggesting F-actin bending explores a distinct structural landscape from the G- to F-transition.

Filament bending deforms protomers

As strain localized to structural elements mediating inter-subunit contacts (Extended Data Fig. 9), we hypothesized that rearrangements at these interfaces could transduce architectural remodelling of the lattice into subunit deformations during filament bending. We therefore examined C_α displacements between bent and helically symmetric models superimposed in the reference frame of individual protomers, which revealed coupling between the reference subunit's deformations and steric encroachment by its neighbors.

In bent ADP-F-actin, protomer 4 longitudinally compresses protomer 2 by wedging its subdomain 3 sites 1 and 2 into protomer 2's nucleotide cleft, pressing down on protomer 2's subdomain 2 core and separating its D-loop from subdomain 4 (Extended Data Fig. 10a, top, Supplementary Video 5). However, in bent ADP-P_i-F-actin, protomers 4's subdomain 3 sites 1 and 2 impinge in a less coordinated manner, diminishing the splitting rearrangement between protomer 2's subdomains 4 and 2. We speculate that this is due to phosphate buttressing against subdomain 2's intrusion into the nucleotide cleft. On the opposite strand, extension of the protomer 1-protomer 3 interface evokes a distinct response (Extended Data Fig. 10a, bottom, Supplementary Video 5). The different direction and smaller magnitude of protomer 3's subdomain 3 site 1 incursion into protomer 1's nucleotide cleft results in negligible displacement of protomer 1's subdomain 4 core. Furthermore, the directional reversal of displacements in protomer 3's subdomain 3 site 2 substantially alters the direction of D-loop repositioning. The presence of phosphate once again alters rearrangements in protomer 1's subdomain 2 core, producing a similar splitting in the displacements of protomer 3 subdomain 3 sites 1 and 2 as that observed on the other strand, thereby modulating rearrangements in protomer 1.

Neighboring protomers also exert steric effects at the lateral interfaces between strands (Extended Data Fig. 10b, Supplementary Video 6). In both ADP- and ADP-P_i-F-actin, the H-plug of subunit 2 is pushed outwards normal to the direction of filament curvature, primarily due to its contact with subdomain 4 from protomer 1. In ADP-P_i-F-actin, protomer 3's subdomain 3 site 1 also contributes to this displacement. Conversely, the H-plug of subunit 3 is pressed in towards the inside of the curve, which is driven by protomer 4's subdomain 3 site 1 in both ADP- and ADP-P_i-F-actin (Extended Data Fig. 10b, bottom). Taken together, these data are consistent with a model in which steric incursions by neighboring protomers elicit lattice-position specific deformations in actin protomers during filament bending, with rearrangements at longitudinal interfaces in particular being modulated by the filament's nucleotide state.

In addition to its coordination with deformations of globular subdomains, we hypothesized the well-established structural flexibility of actin's D-loop[27,32,40,41] could play an important role in facilitating F-

actin bending. We recently reported that a short segment of the D-loop (M47-Q49) adopts a mixture of two conformations in canonical ADP-F-actin, whose prevalence is modulated by myosin motor binding[41]. Examination of the D-loop densities in our bent F-actin maps revealed considerable heterogeneity at this position, with no clear pattern based on a subunit's lattice position or nucleotide state (Extended Data Fig. 9b). However, the density was always well-explained by the previously modelled conformations, suggesting the protomers averaged at these positions in the asymmetric reconstructions randomly sampled the plausible conformational space. This is consistent with M47-Q49 serving as flexible joint which facilitates the maintenance of inter-subunit contacts during mechanical remodelling.
