# Supplementary material for: Bending forces and nucleotide state jointly regulate F-actin structure
Source: Nature. 2022 Oct 26;611(7935):380–6. doi: 10.1038/s41586-022-05366-w (PMC9646526; doi:10.1038/s41586-022-05366-w)
Supplement: Supplementary file 2 — Reporting Summary [file 41586_2022_5366_MOESM2_ESM.pdf]

nature portfolio

Corresponding author(s): Gregory M. Alushin
Last updated by author(s): Sep 13, 2022

Reporting Summary

Nature Portfolio wishes to improve the reproducibility of the work that we publish. This form provides structure for consistency and transparency in reporting. For further information on Nature Portfolio policies, see our Editorial Policies and the Editorial Policy Checklist.

Statistics

For all statistical analyses, confirm that the following items are present in the figure legend, table legend, main text, or Methods section.

n/a	Confirmed	
☐	☒	The exact sample size (*n*) for each experimental group/condition, given as a discrete number and unit of measurement
☒	☐	A statement on whether measurements were taken from distinct samples or whether the same sample was measured repeatedly
☐	☒	The statistical test(s) used AND whether they are one- or two-sided<br>*Only common tests should be described solely by name; describe more complex techniques in the Methods section.*
☒	☐	A description of all covariates tested
☐	☒	A description of any assumptions or corrections, such as tests of normality and adjustment for multiple comparisons
☐	☒	A full description of the statistical parameters including central tendency (e.g. means) or other basic estimates (e.g. regression coefficient) AND variation (e.g. standard deviation) or associated estimates of uncertainty (e.g. confidence intervals)
☐	☒	For null hypothesis testing, the test statistic (e.g. *F*, *t*, *r*) with confidence intervals, effect sizes, degrees of freedom and *P* value noted<br>*Give P values as exact values whenever suitable.*
☒	☐	For Bayesian analysis, information on the choice of priors and Markov chain Monte Carlo settings
☒	☐	For hierarchical and complex designs, identification of the appropriate level for tests and full reporting of outcomes
☒	☐	Estimates of effect sizes (e.g. Cohen's *d*, Pearson's *r*), indicating how they were calculated

Our web collection on statistics for biologists contains articles on many of the points above.

Software and code

Policy information about availability of computer code

Data collection	SerialEM 3.7.0, Nikon NIS-Elements AR 5.21.03
Data analysis	Custom machine learning software for cryo-EM analysis: https://github.com/alushinlab/bent_actin; python scripts for analyzing TIRF movies: https://doi.org/10.5281/zenodo.6929148. CryoSPARC 2.11.0, EMAN2.22, RELION-3.1, RELION-3.0, UCSF Chimera 1.12, UCSF ChimeraX 1.2.4, python 2.7.14, TensorFlow 1.5.0, scikit-image 0.14.2, cryoDRGN 0.3.1, ISOLDE 1.2.0, COOT 0.9.1, Phenix 1.19.2-4158, resample.exe from FREALIGN v9.11, MotionCor2 1.3.2, CTFFIND 4.1.5, CASTp 3.0, Matplotlib 2.2.3, GraphPad Prism 9.4.1

For manuscripts utilizing custom algorithms or software that are central to the research but not yet described in published literature, software must be made available to editors and reviewers. We strongly encourage code deposition in a community repository (e.g. GitHub). See the Nature Portfolio guidelines for submitting code & software for further information.

Data

Policy information about availability of data

All manuscripts must include a data availability statement. This statement should provide the following information, where applicable:

- Accession codes, unique identifiers, or web links for publicly available datasets
- A description of any restrictions on data availability
- For clinical datasets or third party data, please ensure that the statement adheres to our policy

Cryo-EM density maps and corresponding atomic models have been deposited in the PDB and EMDB with the following accession codes: Helically-symmetric ADP-F-

actin (PDB: 8D13, EMDB: EMD-27114); helically-symmetric ADP-Pi-F-actin (PDB: 8D14, EMDB: EMD-27115); asymmetric bent ADP-F-actin (PDB: 8D15, EMDB: EMD-27116); asymmetric bent ADP-Pi-F-actin (PDB: 8D16, EMDB: EMD-27117); asymmetric straight ADP-F-actin control 1 (PDB: 8D17, EMDB: EMD-27118); asymmetric straight ADP-F-actin control 2 (PDB: 8D18, EMDB: EMD-27119). Cryo-EM datasets have been deposited in the EMPIAR with the following accession codes: ADP-F-actin (EMPIAR-11128); ADP-Pi-F-actin (EMPIAR-11129). These depositions include the raw movies and processed particle stacks used to generate the final reconstructions deposited in the EMDB. Datasets for cryoDRGN analysis, neural network training, and cofilin severing assays are available through Zenodo. Synthetic datasets used to train denoising auto-encoder and semantic segmentation neural networks as well as the trained networks are accessible at https://doi.org/10.5281/zenodo.6917913. CryoDRGN reconstructions, fitted models, trained cryoDRGN networks, and the data required to train the cryoDRGN networks are accessible at https://doi.org/10.5281/zenodo.6928604. Cofilin TIRF microscopy data are accessible at https://doi.org/10.5281/zenodo.6929148. Source data are provided with this paper. All other data required to assess this study's conclusions are presented in the manuscript.

Human research participants

Policy information about studies involving human research participants and Sex and Gender in Research.

Reporting on sex and gender	N/A
Population characteristics	N/A
Recruitment	N/A
Ethics oversight	N/A

Note that full information on the approval of the study protocol must also be provided in the manuscript.

Field-specific reporting

Please select the one below that is the best fit for your research. If you are not sure, read the appropriate sections before making your selection.

☒ Life sciences ☐ Behavioural & social sciences ☐ Ecological, evolutionary & environmental sciences

For a reference copy of the document with all sections, see nature.com/documents/nr-reporting-summary-flat.pdf

Life sciences study design

All studies must disclose on these points even when the disclosure is negative.

Sample size	Sample sizes were not predetermined in our experimental design. TIRF assays were performed 3-4 times (exact n indicated in Extended Data Fig. 1 legend), as is standard practice for in vitro biochemical assays, with consistent results. For cryo-EM experiments, thousands of micrographs and hundreds of thousands of filament segments were analyzed (see Methods and Supplementary Table 1). The minimal sample size was determined to be that which resulted in consistent, high-resolution reconstructions. Multiple independently analyzed random subsets from each condition converged to the same three-dimensional structure, confirming this was achieved.
Data exclusions	No data were excluded.
Replication	3-4 independent TIRF assays were performed for each condition (exact n indicated in Extended Data Fig. 1 legend). Cryo-EM datasets were collected in single multi-day sessions from individual specimens, as is customary in our field, and thus they were not directly replicated. However, the atomistic structural similarity between independently prepared and imaged ADP- and ADP-Pi-F-actin (Fig. 1) supports the reproducibility of our structure determination pipeline.
Randomization	For cryo-EM structural analysis, particles were randomly assigned to independent half datasets for resolution analysis. Randomization was otherwise not relevant for our study, which did not employ live animals or human subjects.
Blinding	Blinding was not relevant for our study, as it did not employ live animals or human subjects.

Reporting for specific materials, systems and methods

We require information from authors about some types of materials, experimental systems and methods used in many studies. Here, indicate whether each material, system or method listed is relevant to your study. If you are not sure if a list item applies to your research, read the appropriate section before selecting a response.

Materials & experimental systems

n/a	Involved in the study	
☒	☐	Antibodies
☒	☐	Eukaryotic cell lines
☒	☐	Palaeontology and archaeology
☒	☐	Animals and other organisms
☒	☐	Clinical data
☒	☐	Dual use research of concern

Methods

n/a	Involved in the study	
☒	☐	ChIP-seq
☒	☐	Flow cytometry
☒	☐	MRI-based neuroimaging
